# *QuickStats:* Age-Adjusted Percentage* of Adults Aged 25–64 Years Who Are Very Worried About Their Ability to Pay Medical Bills if They Get Sick or Have an Accident,^†^ by Sex and Veteran Status — National Health Interview Survey, United States, 2019^§^

**DOI:** 10.15585/mmwr.mm7015a8

**Published:** 2021-04-16

**Figure:**
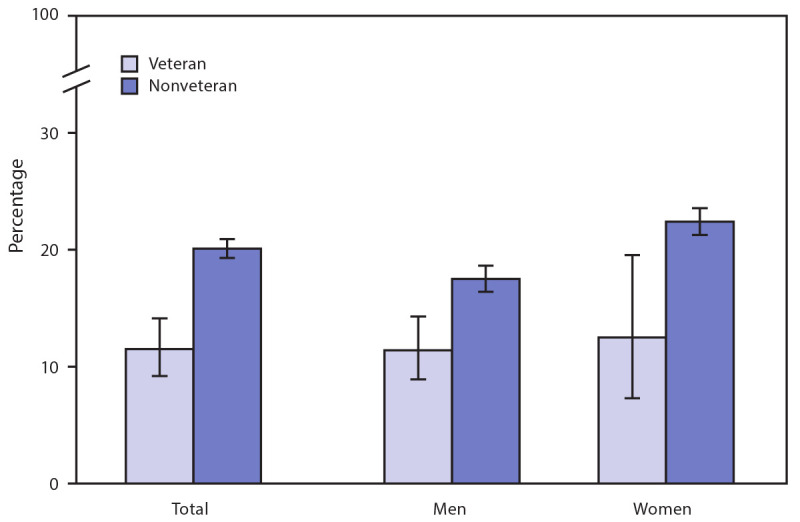
In 2019, among adults aged 25–64 years, veterans (11.5%) were less likely than nonveterans (20.1%) to be very worried about their ability to pay their medical bills if they get sick or have an accident. This pattern was found for both men and women, with veterans less likely than nonveterans to be very worried about medical bills: 11.4% versus 17.5% for men and 12.5% versus 22.4% for women, respectively.

**Source:** National Center for Health Statistics, National Health Interview Survey, 2019 data. https://www.cdc.gov/nchs/nhis.htm

